# ZnO Semiconductor Nanoparticles and Their Application in Photocatalytic Degradation of Various Organic Dyes

**DOI:** 10.3390/ma14247537

**Published:** 2021-12-08

**Authors:** Priscy Alfredo Luque-Morales, Alejandra Lopez-Peraza, Osvaldo Jesus Nava-Olivas, Guillermo Amaya-Parra, Yolanda Angelica Baez-Lopez, Victor Manuel Orozco-Carmona, Horacio Edgardo Garrafa-Galvez, Manuel de Jesus Chinchillas-Chinchillas

**Affiliations:** 1 Facultad de Ingeniería, Arquitectura y Diseño, Universidad Autónoma de Baja California (UABC), Ensenada 22860, Baja California, Mexico; pluque@uabc.edu.mx (P.A.L.-M.); alejandra.lopez.peraza@uabc.edu.mx (A.L.-P.); navao@uabc.edu.mx (O.J.N.-O.); amaya@uabc.edu.mx (G.A.-P.); yolanda@uabc.edu.mx (Y.A.B.-L.); horacio.garrafa@uabc.edu.mx (H.E.G.-G.); 2 Centro de Investigación en Materiales Avanzados (CIMAV), Chihuahua 31136, Chihuahua, Mexico; 3 Departamento de Ingeniería y Tecnología, Universidad Autónoma de Occidente (UAdeO), Guasave 81048, Sinaloa, Mexico

**Keywords:** biosynthesis, *Capsicum annuum var. Anaheim*, organic dye, photocatalysis, ZnO nanoparticles

## Abstract

The biosynthesis of oxide semiconductor nanoparticles (NPs) using materials found in nature opens a wide field of study focused on sustainability and environmental protection. Biosynthesized NPs have the capacity to eliminate organic dyes, which pollute water and cause severe damage to the environment. In the present work, the green synthesis of zinc oxide (ZnO) NPs was carried out using *Capsicum annuum var. Anaheim* extract. The photocatalytic elimination of methylene blue (MB), methyl orange (MO), and Rhodamine B (RhB) in UV radiation was evaluated. The materials were characterized by scanning and transmission electron microscopy (SEM and TEM) and SEM-coupled energy dispersive spectroscopy (EDS), attenuated total reflectance-infrared (ATR-IR), X-ray photoelectron spectroscopy (XPS), X-ray diffraction (XRD), Photoluminescence (PL), and ultraviolet-visible spectroscopy (UV-Vis). The TEM analysis showed the NPs have an average size of 40 nm and quasi-spherical shape. ATR-IR showed the ZnO NPs contained functional groups from the extract. The analysis through XRD indicated that the NPs have a hexagonal zincite crystal structure with an average crystallite size of approximately 17 nm. The photoluminescence spectrum (PL) presented an emission band at 402 nm. From the UV-Vis spectra and TAUC model, the band-gap value was found to be 2.93 eV. Finally, the photocatalytic assessment proved the ZnO NPs achieved 100% elimination of MB at 60 min exposure, and 85 and 92% degradation of MO and RhB, respectively, at 180 min. This indicates that ZnO NPs, in addition to using a friendly method for their synthesis, manage to have excellent photocatalytic activity in the degradation of various organic pollutants.

## 1. Introduction

Environmental pollution is a topic of interest to the scientific community, as the increase in damage caused to ecosystems is becoming more noticeable. Water is an essential natural resource for life on the planet; however, the lack of awareness from society and the high percentage of pollution in water systems (rivers, seas, streams, etc.) brought about by industrial processes generates eutrophication, consumes oxygen from water and causes the death of living species that inhabit these ecosystems (flora and fauna) [1]. Therefore, it is important to deepen research to minimize these problems, or to find alternatives to eliminate pollutants (environmental remediation) [2]. Within the wide classification of waste generated by industries around the world, organic dyes are of great concern, as they are resistant to biodegradation and prevent the penetration of sunlight into water, causing negative effects on photosynthesis [3]. Furthermore, organic dyes have chemical compositions that, for the most part, have turned out to be very stable, resistant, toxic, potentially mutagenic, and carcinogenic [4]. These dyes are used in the pharmaceutical, food, cosmetic, plastic, paper, and textile industries. The textile industry causes the highest levels of water pollution. The existence of around 10,000 colorants has been reported, of which approximately 700,000 tons are generated per year [5,6,7]. Some techniques used for the elimination of organic dyes, pollutants, and non-biodegradable residues in water are ion exchange, adsorption, ozonation, Fenton reaction, reductive degradation, and microbial processes, among others [8]. However, some of these techniques are expensive, use dangerous reagents, generate toxic by-products, and do not eliminate the source of the problem [9]. Amid the techniques used, photocatalysis is a simple, low-cost process that does not cause toxic by-products. This technique has been shown to be highly effective in degrading pollutants in water using solar energy or UV energy. In 2021, J. Yin et.al. synthesized polyvinylidene fluoride (PVDF) and titanium dioxide (TiO_2_) membranes for the photocatalytic degradation of MO, achieving 95% degradation in 200 min [10]. Furthermore, Al-Gharibi et.al. synthesized a silver nanoparticle (Au-NPs) and zinc oxide nanorod (ZnO-NRs) composite, and used it with sunlight-assisted photocatalysis to degrade 92% of contaminating paracetamol within 4 h [11].

In the photocatalytic process, a photocatalyst is used, which generally is a semiconductor material that, by absorbing photons, is activated to produce hydroxyl radicals (oxidant) and thus helps the total degradation of organic dyes [12]. The photocatalysts mostly used today are oxide semiconductor NPs, such as titanium dioxide (TiO_2_), tin dioxide (SnO_2_), copper oxide (CuO), iron oxide (III) (Fe_2_O_3_), wolfram oxide (WO_3_), ZnO, etc. [13,14,15,16,17,18]. ZnO is a material with excellent thermal stability and is a multifunctional material. It has been used in applications such as luminescent material, sensors, solar cells, batteries, optoelectronic applications and with good results in photocatalysis; in fact, it is considered one of the best photocatalysts for the degradation of pollutants in water [19]. ZnO NPs have a favorable band-gap of around 3.37 eV, have strong oxidizing and non-toxic nature, excellent chemical and mechanical stability, and have shown to be photosensitive, leading to their use in various applications [20]. There are various techniques for the synthesis of these NPs, some related to the top-down approach (atomization, laser ablation, radiofrequency, sputtering, etc.) and others related to the bottom-up approach (sol gel process, template synthesis, electro-deposition, microwave-assisted synthesis, etc.) [21,22,23,24]. Unfortunately, most of these techniques impose a series of drawbacks, such as high cost, use of organic solvents, toxic stabilizing agents, toxic by-products, and most are highly reactive and harmful to the environment [25]. The current research challenge is to apply synthesis methods that are more environmentally friendly, and to use them to obtain oxide semiconductor-based NPs. One such method is green synthesis, which consists in using materials found in nature (biologic materials). Such methods help to improve processes that are compatible with the environment, makes them cheaper, safer, faster, simpler, use ecologic agents, and produce fewer harmful by-products [26]. Among the investigations that have reported the use of green synthesis are those by P.A. Luque, et.al. in 2021, where he used *Camellia sinensis* to obtain SnO_2_ NPs of 4.7 nm particle size [27]. On the other hand, Ghulam Nabi et.al., in 2020, used lemon peel extract to biosynthesize TiO_2_ NPs, achieving particle sizes of 80–140 nm, and quasi-spherical shape [28]. Furthermore, K. Velsankar, et.al., in 2020, used *Allium sativum* extract for the biosynthesis of CuO NPs, achieving particle sizes of 20–40 nm [29]. Furthermore, biosynthesized NPs have already been used for the photocatalytic degradation of organic dyes. M. Aravind et.al. synthesized Au-NPs using Jasmine flower extract in 2021, and used them to degrade 78% of contaminating MB within 120 min [30]. On the other hand, L. Ardakani et.al. synthesized iron NPs (Fe-NPs) using *Chlorophytumcomosum* extract in 2021, presenting a 77% degradation of MO within 6 h [31]. S. Rajendrachari et.al., also in 2021, used cauliflower to biosynthesize ZnO NPs, managing to degrade 75% RhB within 2 h [32]. As has been disclosed in the literature, various natural stabilizing agents have been used, such as roots, leaves, fruits, and vegetables [33]; however, what has been seldom studied is the use of chili peppers in the green synthesis of ZnO NPs. Specifically, the use of Anaheim Chili (*Capsicum annuum var. Anaheim*) has not been reported on for the biosynthesis of NPs. This material contains flavonoids, ascorbic acid, polyphenols, and capsaicin in its molecular composition, which makes it an important candidate to be used in the green synthesis of NPs [34,35]. In this research, the green synthesis of ZnO NPs was carried out using an extract of *Capsicum annuum var. Anaheim*. The biosynthesized NPs were characterized by SEM/EDS, TEM, ATR-IR, XRD, PL and UV-Vis. In addition, through UV-Vis, the photocatalytic activity of these NPs was evaluated for the elimination of three pollutants in water (MB, MO and RhB).

## 2. Materials and Methods

### 2.1. Materials

For the green synthesis, the following were used: fresh Anaheim chili (*Capsicum annuum var. Anaheim*) acquired locally; 98% pure zinc nitrate (Zn (NO_3_)_2_•6H_2_O) purchased from Sigma Aldrich, Toluca, México (CAS number 10196-18-6); deionized water with a pH of 7.2 ± 0.2. For the photocatalytic activity, three organic dyes acquired from Fagalab, Mocorito, México were used: MB (Mw. Of 373.9 g/mol, 99% purity, CAS number 61-73-4); MO (Mw. 327.34 g/mol, 95% purity, CAS number 547-58-0); RhB (Mw. Of 479.01 g/mol, 95% purity, CAS number 81-88-9).

### 2.2. Extract Preparation

For the preparation of the *Capsicum annuum var. Anaheim* extract, 1 g of chili was added to 50 mL deionized water and stirred for 2 h at room temperature. Subsequently, the solution was placed in a thermal bath for 1 h at 60 °C. Finally, the solution was filtered with a vacuum pump using a #4 Whatman filter. The extract was stored to be use later on in the biosynthesis process.

### 2.3. Green Synthesis of ZnO NPs

For the biosynthesis of the ZnO NPs, 2 g of Zn(NO_3_)_2_•6H_2_O was added to 50 mL of *Capsicum annuum var. Anaheim* extract, and subjected to magnetic stirring in darkness for 1 h at room temperature. Subsequently, the solution was placed in thermal bath for 16 h at 60 °C. After that time, the solution acquired a plastic consistency. The sample was placed in a porcelain capsule and heat-treated in a Thermolyne oven, model Furnace 48000, in an air environment, with a heating gradient of 100 °C/5 min up to 400 °C; it was further treated at this temperature for an additional hour. Finally, the resulting material was cooled down, manually ground, and stored.

### 2.4. Photocatalytic Activity

For the photocatalytic activity assessment, three solutions corresponding to the three organic dyes being studied were prepared to a concentration of 15 mg/L in 50 mL of deionized water. Subsequently, 50 mg of photocatalyst (ZnO NPs) were added to each of the contaminated solutions (1:1 ratio), and they were stirred in darkness for 30 min until adsorption–desorption equilibrium was reached. Afterwards, the solutions were placed in reactors with UV light lamps (Polaris UV-1C, equipped with a 10 W bulb and 18 mJ/cm^2^ energy) to monitor dye degradation during 3 h of exposure time. Over the course of the first hour, samples were obtained every 10 min. During the second hour, degradation was evaluated every 20 min. Finally, over the third hour of study, the samples were analyzed every 30 min.

### 2.5. ZnO NPs Characterization

The morphology of the ZnO NPs was analyzed via SEM using a JEOL JSM-5300 scanning electron microscope, at a working distance of 11 mm and a voltage of 15 kV. The elemental composition was performed via EDS coupled to the SEM equipment, using Aztec software (Oxford). The TEM analysis was performed with a JEOL JEM-2010F transmission electron microscope, with an acceleration of 120 kV. The structural analysis of the ZnO NPs was performed using an ATR-IR piece of equipment (Perkin Elmer Brand, 0.5 cm^−1^ resolution and 400 to 3500 cm^−1^ measurement range) and via XRD (Bruker-D2 Phase, with a radiation of Cu Ka = 1.541 Å at a step of 0.022 from 10 to 80°). For the elemental chemical analysis, an XPS system (Axis-Ultra, Kratos, in SPECS system using Al Kα monochromatic X-rays at 1486.6 eV) was used. To evaluate the optical properties of the NPs, the PL (Horiba Nanlog, using ethanol as solvent and a concentration of 100 ppm) and UV-Vis (Perkin Elmer brand spectrophotometer, Lambda 365 with a wavelength of 190–800 nm, and a scanning speed of 600 nm/s) spectra were analyzed.

## 3. Results and Discussion

### 3.1. Nanoparticles

#### 3.1.1. SEM/EDS

**Figure 1:**
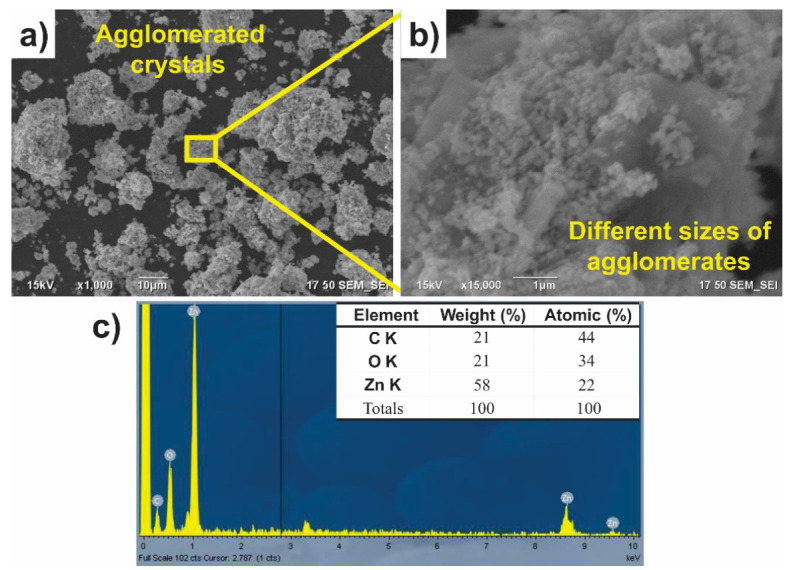
SEM/EDS of ZnO NPs biosynthesized with *Capsicum annuum var. Anaheim.* (**a**) and (**b**) Agglomeration of nanoparticles and (**c**) EDS analysis.

The morphology of ZnO NPs biosynthesized with *Capsicum annuum var. Anaheim* is observed in Figure 1, where certain crystal agglomerates separated from each other are observed, but, when zooming in on the micrograph (Figure 1b), it is possible to observe the different zones of agglomerates of ZnO NPs of different sizes and quasi-spherical shape. This is an indication of the successful formation of the nanomaterial. Morphologies very similar to those presented in this investigation have been reported in the literature [36,37]. In addition, the EDS analysis in Figure 1c depicts the percentage of the elements present in the NPs. As observed in the results, the only elements found in the material were C, O, and Zn, which correspond to elements present in the organic molecules of the extract and to the zinc oxide NPs [38].

#### 3.1.2. TEM

**Figure 2:**
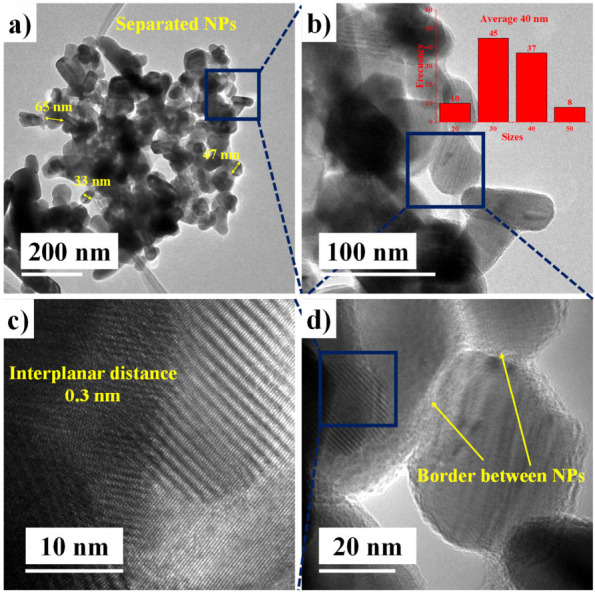
TEM analysis of ZnO NPs biosynthesized with *Capsicum annuum var. Anaheim.* (**a**) Morphology of the NPs, (**b**) size distribution, (**c**) interplanar distance and (**d**) border between NPs.

The morphology of the ZnO NPs biosynthesized by *Capsicum annuum var. Anaheim* is seen in Figure 2. Figure 2a shows that NPs have a quasi-spherical shape and showed different growths. There are NPs of different sizes (65, 47, 33 nm etc.) and shapes (circular, oval, elongated, irregular, etc.). The measurement frequency presented a nanoparticle average size of 40 nm in 100 measurements, where sizes ranging between 20 and 50 nm were recorded (Figure 2b). A very similar size has previously been reported for ZnO NPs biosynthesized with another stabilizing agent [39,40]. By zooming in on the image (Figure 2c), the boundaries between the biosynthesized NPs become distinguishable. This border or edge demonstrates the separation of NPs, which denotes that the stabilizing agent accomplished its purpose. Additionally, in Figure 2d, the fingerprint crystalline planes can be observed and the interplanar distance can be measured, which oscillates around 0.3 nm. This distance was measured between the planes that correspond to (100), which was verified by XRD [41].

#### 3.1.3. ATR-IR

**Figure 3:**
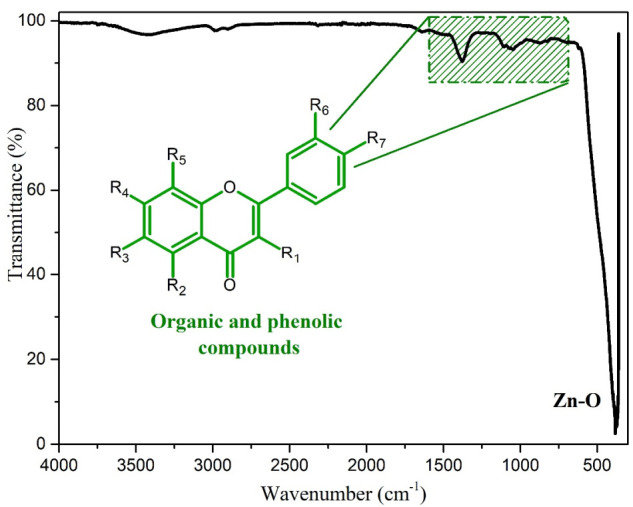
ATR-IR of ZnO NPs biosynthesized with *Capsicum annuum var. Anaheim*.

The ATR-IR spectrum of ZnO NPs biosynthesized with *Capsicum annuum var. Anaheim* is shown in Figure 3. The analysis was performed in a frequency range of 4000–300 cm^−1^, at room temperature. In the spectrum, different bands can be identified, which are found in the region of 1600–800 cm^−1^; These bands correspond to the organic content and phenolic groups within the samples belonging to the extracts of *Capsicum annuum var.* *Anaheim* [42]. The bands observed at 861 cm^−1^ could be assigned to the functional groups C–H (aromatic). The absorption peak at 1378 cm^−1^ can be attributed to the vibration of the C-N bonds of the alkaloids present in the Anaheim pepper [43]. The spectrum presented a band around 380 cm^−1^; this signal belongs to the Zn–O bond, characteristic of ZnO NPs, which confirms the successful synthesis of ZnO NPs [44].

#### 3.1.4. XPS

**Figure 4:**
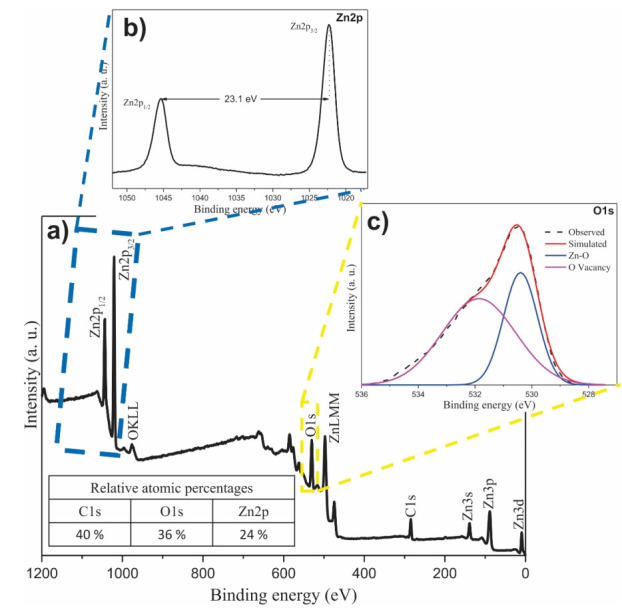
XPS analysis of ZnO NPs biosynthesized with *Capsicum annuum var. Anaheim*. (**a**) General spectrum (Inset: table of atomic percentages), (**b**) high resolution of O1s, and (**c**) high resolution of Zn2p.

To know the elemental composition and the chemical environment of the biosynthesized NPs, an XPS analysis was carried out, which is shown in Figure 4. The energy of the spectra was calibrated with respect to the main peak, C1s at 284.5 eV [45]. The result of the analysis of the general spectrum is shown in Figure 4a, in the general spectrum you can see the main peaks C1s (284.5 eV), O1s (530.5 eV), and Zn2p (1022.3 and 1045.4 eV for Zn2p_3/2_ and Zn2p_1/2_, respectively). The appearance of the main C1s peak confirms the presence of the organic molecules of the extract, as has been found in reports where plant extracts are used [46], while the presence of the O1s and Zn2p peaks are due to the nanoparticles [47]; the existence of only these main peaks confirms what was found by EDS, which was additionally proven by calculating the atomic percentages, equivalent to those found by the EDS technique. The percentages obtained are denoted in the inset table in Figure 4a. These results demonstrate the obtaining of ZnO NPs together with the presence of organic molecules from the *Capsicum annuum var. Anaheim* extract. In addition, high resolution analysis was performed for the Zn2p and O1s peaks, which are shown in Figure 4b,c, respectively. For the Zn2p peak, a doublet was found consisting of the signals Zn2p_1/2_ and Zn2p_3/2_, with an energy difference of 23.1 eV between both signals. This energy difference belongs to the Zn^2+^ species which is the characteristic species of Zn in ZnO NPs [48], indicating its synthesis. In the case of the O1s peak, whose maximum intensity was found at 530.5 eV, it was deconvolved into 2 signals, of which the signal that appears at 530.4 eV belongs to the Zn-O bond; owing to the bond in the ZnO NPs. While the other signal, at 531.9 eV, belongs to oxygen vacancies [49,50]. These vacancies are generated by the presence of molecules from the organic material of the *Capsicum annuum var. Anaheim* extract.

#### 3.1.5. XRD

**Figure 5:**
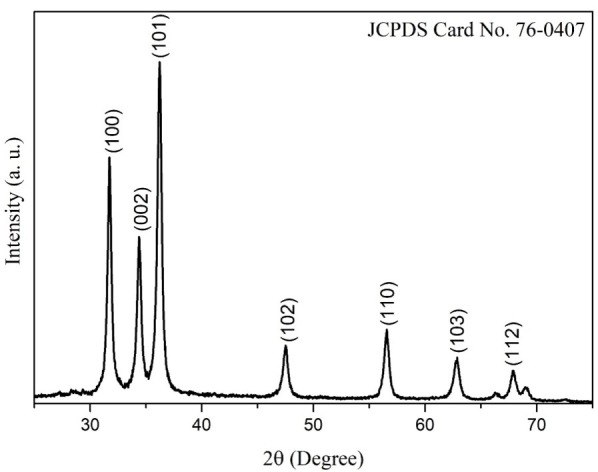
XRD of ZnO NPs biosynthesized with *Capsicum annuum var. Anaheim*.

Figure 5 shows the XRD analysis of the biosynthesized ZnO NPs. The characteristic peaks of this material are clearly observed in the diffraction pattern, identifying them at 31.74, 34.38, 36.22, 47.55, 56.54, 62.81, and 67.85° 2θ, corresponding to the Miller indexes of (100), (002), (101), (102), (110), (103), and (112), respectively. These peaks coincide with those of the JCPDS Card No. 76-0704, which describes the ZnO NPs as hexagonal structures type zincite [43]. No extra peaks were found, indicating there are no impurities. To know the crystallite size, the Debye–Scherrer formula (Equation (1)) was used in the three most intense peaks (100), (002) and (101):

(1) $$L=\frac{K\lambda }{\beta \cos\theta }$$

where L is the dimension measure of the particles, 𝜆 the incident wavelength, β the full width at half maximum of the peak, θ the Bragg angle, and K takes the value of 0.9 [51]. The results showed that the biosynthesized NPs had an average crystallite size of 17 nm. Different values from TEM analysis, because crystallite size is a coherent diffraction domain and is not the same as particle size (TEM), due to the presence of polycrystalline and mono-crystalline aggregates [52]. The size obtained in this study is within the range of previous reports in the literature for ZnO NPs [21,53].

In this work, Rietveld refinement was performed with High Score Plus software on XRD patterns. The results of the Rietveld refinement disclosed a zincite phase of a hexagonal structure with crystallite strain of 0.38 and cell volume of 47.81 Å^3^, obtaining a goodness of fit (GOF) adjustment of 2.88. The parameters obtained coincide with that reported in literature [54,55], these results confirm the obtaining of the NPs of ZnO in zincite phase. Table 1 shows the parameters obtained from Rietveld refinement.

#### 3.1.6. PL

**Figure 6:**
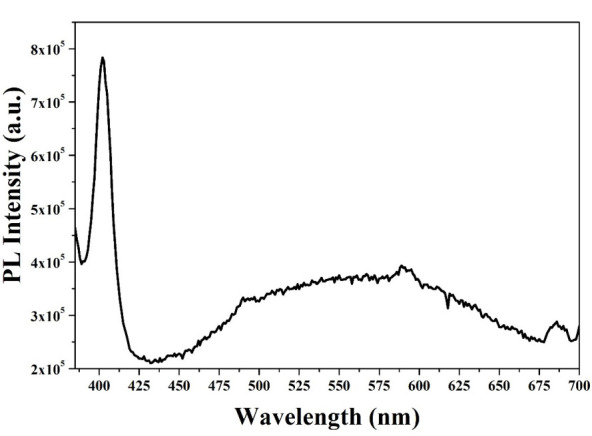
PL spectrum of ZnO NPs biosynthesized with *Capsicum annuum var. Anaheim*.

The spectrum of PL was performed in a wavelength range from 385 to 700 nm with a λ_ex_ of 350 nm, as is observed in Figure 6. A sharp and well-defined ultraviolet emission can be observed in the spectrum, and a very wide visible emission. This is due to the microstructure of ZnO NPs, since a change in particle size is related to the change in optical properties [56]. At approximately 400 nm, a peak that corresponds to the emission of the near band edge is observed, which is attributed to the direct recombination of free excitons in the ZnO NPs. On the other hand, in the visible emission zone (425 to 675), the emission of defects in the ZnO network occurs, such as interstitial defects of Zn and oxygen vacancies [57].

#### 3.1.7. UV-Vis (Band-Gap)

By studying the absorbance of the biosynthesized ZnO NPs, assayed in the UV-Vis spectrum, the energy required to make an electron jump from its valence band to the conduction band in these NPs was determined. The band-gap study was carried out following the TAUC model (Equation (2)).

**Figure 7:**
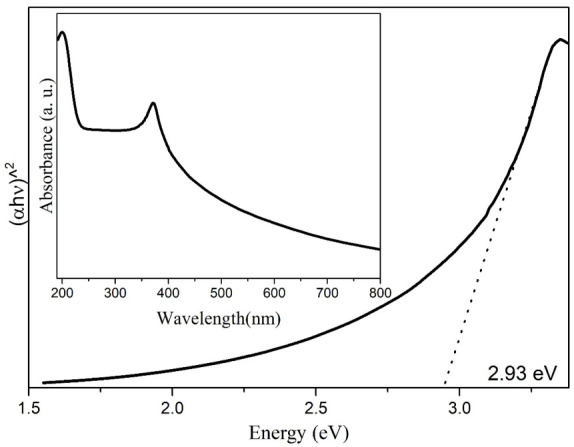
TAUC graph (Inset: UV-Vis absorbance spectrum) of ZnO NPs biosynthesized with *Capsicum annuum var. Anaheim*.

(2) ∝hν1n=Bhν−Eg

where α (ν) corresponds to the absorption coefficient (Lambert–Beer), hν is equal to the energy of the incident photon, B is a constant, E_g_ is the band-gap energy, and, finally, the value of “n” corresponds to the type of electronic transition n = 1/2 [58]. The value depicted/from the band-gap corresponding to the *Capsicum annuum var. Anaheim* biosynthesized ZnO NPs was 2.93 eV, which can be seen in Figure 7, lower than the value reported for conventional ZnO NPs [59]. This means the constituents of *Capsicum annuum var. Anaheim* extract work as photosensitizers [60], which reveals that they can be used in photocatalysis processes to eliminate contaminants in water. Similar values have been previously reported in the literature [61]. The absorbance spectrum of the biosynthesized ZnO NPs can be observed in the inset of Figure 7, which presents a band with λ_max_ at 370 nm. This band has been assigned in previous reports as characteristic of ZnO NPs [62,63]. As in ATR-IR and XRD, this indicates a successful synthesis of ZnO NPs.

### 3.2. Formation Mechanism

**Figure 8:**
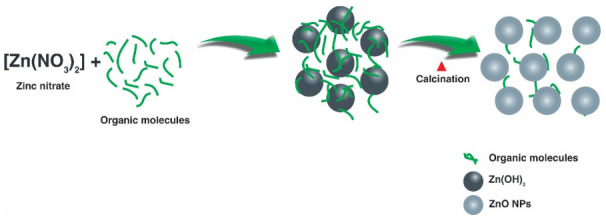
Proposed formation mechanism for ZnO NPs biosynthesized with *Capsicum annuum var. Anaheim*.

Based on the characterization studies carried out on the biosynthesized NPs, a proposal for the formation mechanism of the NPs is made. The formation scheme is shown in Figure 8. Considering that the extract of *Capsicum annuum var. Anaheim* is a source rich in vitamins, carotenoids, flavonoids, etc. [64], the most accepted mechanism suggests that, when zinc nitrate is in solution with the extract, the Zn^2+^ ions are uniformly distributed [65]. In the process, as the Zn^2+^ ions are in direct interaction with the OH groups of the biomolecules in the extract, the biomolecules work as electron donors, donating their electrons to the electrophilic Zn^2+^ species, leading to the oxidation of the hydroxyl groups and the reduction of the Zn^2+^ ions [66]. Through this interaction, a link is generated between the Zn^2+^ ions and the biomolecules to form a stable complex [67]. Subsequently, the formation of ZnO NPs occurs when subjected to heat treatment [68]. The formation of the ZnO NPs by the effect of the biomolecules within the extract indicates that the biomolecules act as capping and chelating agents [69].

### 3.3. Photocatalytic Activity

**Figure 9:**
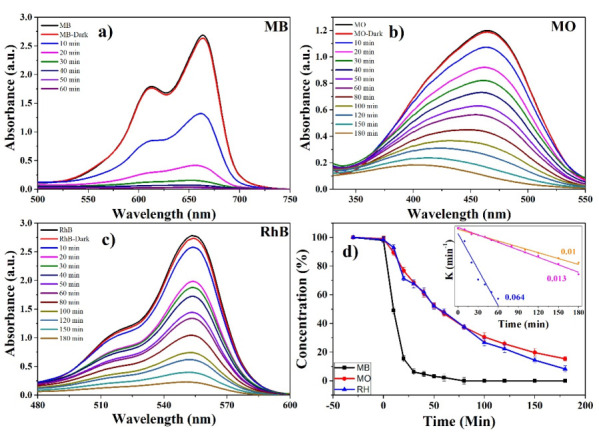
Photocatalytic study: (**a**) UV-Vis of MB, (**b**) UV-Vis of MO, (**c**) UV-Vis of RhB and (**d**) complete study of degradation of organic dyes using ZnO NPs biosynthesized with *Capsicum annuum var. Anaheim*.

In addition to using an ecological route for the synthesis of ZnO NPs, its application in the elimination of pollutants in water significantly helps to solve environmental problems worldwide. The photocatalytic study in the elimination of three organic dyes (MB, MO and RhB) is observed in Figure 9. The absorption bands of greater intensity of MB, MO, and RhB are found at 664, 470, and 550 nm, respectively [70,71]. The initial concentration of the pollutants marked the starting point of the measurements. It should be noted that, in Figure 9a–c, as the exposure time in UV light increases, the concentration decreases markedly. On the other hand, Figure 9d presents the complete degradation study, showing that the three dyes presented as light decrease in concentration after 30 min of stirring in darkness (approximately 1%). This occurs by the adsorption process due to NPs-dye affinity [20]. These graphs show that the photocatalyst accelerated the MB degradation process, achieving 100% degradation in approximately one hour of exposure (60 min). In 180 min, 85% of the MO was degraded. For the RhB pollutant, its degradation was 92% in 180 min. This data corroborates the inset shown in Figure 9d, which depicts the highest degradation rate constant, 0.064, as that of the MB solution. It is reported in the literature that natural degradation of these pollutants is around several hours of exposure [72,73,74]. Additionally, Table 2 shows other results reported in the literature. This indicates that the *Capsicum annuum var. Anaheim* biosynthesized ZnO NPs are very useful for accelerating the degradation of these organic colorants in water.

**Figure 10:**
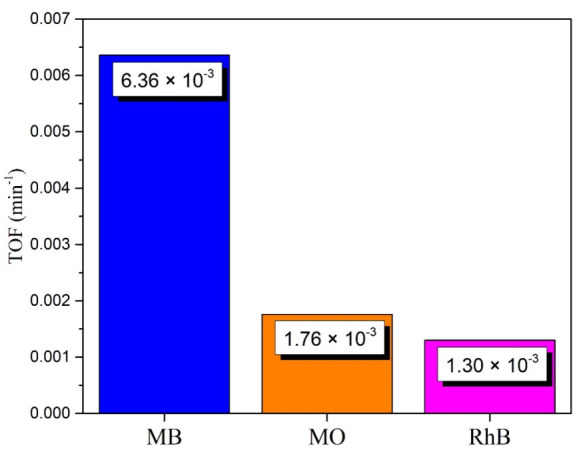
TOF achieved in photocatalytic degradation of MB, MO, and RhB using ZnO NPs biosynthesized with *Capsicum annuum var. Anaheim*.

For a more in-depth analysis of the photocatalytic degradation process for the three colorants, the turnover frequency (TOF) calculation was performed to estimate the number of molecules that were degraded, as well as the number of catalytic cycles during photocatalytic degradation per unit of time [27]. The TOF was obtained with the model of Equation (3):

(3) $$\mathrm{TOF}=\frac{\left(\mathrm{conversion}\%\right)\left(\mathrm{Number}\mathrm{of}\mathrm{moles}\mathrm{of}\mathrm{substrate}\right)}{\left(\mathrm{Number}\mathrm{of}\mathrm{moles}\mathrm{of}\mathrm{catalyst}\right)\left(\mathrm{Time}\right)}$$

where the conversion % is the degradation achieved by the photocatalyst. The results obtained are shown in Figure 10, where values of 6.36 × 10^−3^, 1.76 × 10^−3^, and 1.30 × 10^−3^ for MB, MO, and RhB, respectively, can be observed. The highest TOF value achieved is that for MB, which is several times higher than the MO and RhB values, likewise regarding the degradation constants k. These values are outstanding in comparison with other photocatalytic systems reported in literature [80,81,82].

### 3.4. Photodegradation Mechanism

**Figure 11:**
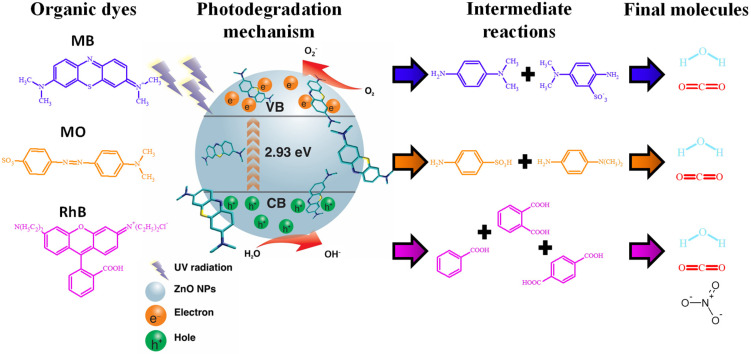
Photocatalytic degradation mechanism of MB, MO and RhB.

Figure 11 describes a degradation mechanism of the dyes analyzed in this investigation, MB, MO, and RhB. The degradation of the dyes begins with the adsorption of the dye on the surface of the NPs due to the affinity that exists between them [83]. Once the molecules have been adsorbed, they are irradiated with ultraviolet light between 3.1 and 6.2 eV; an energy greater than the band-gap of the ZnO NPs biosynthesized with *Capsicum annuum var.* (2.93 eV). With this irradiation, an electron excitation occurs in the valence band (VB), which subsequently passes to the conduction band (CB), giving rise to the generation of an electron-hole pair [76]. When this occurs, the photoexcited electrons interact with molecular oxygen (O_2_), generating superoxide radicals ($O_{2}^{*}$), while the holes interact with water molecules to generate hydroxyl radicals (${\mathrm{OH}}^{-}$) [84]. The $O_{2}^{*}$ and ${\mathrm{OH}}^{-}$ radicals are both species with very high oxidation coefficients [85]; therefore, when these radicals interact with the molecules of the dyes, they cause the degradation of the initial molecules generating by-products. The proposal shown in Figure 11 was made based on the literature. In the case of MB, the $O_{2}^{*}$ and ${\mathrm{OH}}^{-}$ radicals interact with the S atoms of the $C-S^{+}=C$ group and reduce the C=N bond. Subsequently, some demethylation and dechlorination processes occur, obtaining CO_2_, H_2_O, and some mineral species as by-products [86,87,88]. On the other hand, the photocatalytic degradation of MO begins with the breaking of the N=N bond, causing the separation of the molecule. The radicals then attack the CH_3_-N-CH_3_ group, decomposing the CH_3_ group. Subsequently, one of the aromatic rings is released, and some intermediate steps take place, to generate the final by-products, such as CO_2_ and H_2_O [89,90,91]. Finally, the RhB degradation process begins when the double bond of the unsaturated chromophore is attacked by the $O_{2}^{*}$ and ${\mathrm{OH}}^{-}$ radicals until it is broken. Subsequently, there is the consecutive elimination of the ethyl groups, and other intermediate steps, such as the opening of aromatic rings and mineralization. In the end, CO_2_ and H_2_O are obtained [92,93,94].

## 4. Conclusions

Obtaining ZnO NP through green synthesis using Anaheim chili (*Capsicum annuum var. Anaheim*) was successful. Using this ecological method, ZnO NPs were obtained with an average size of 40 nm, a zincite-type crystalline structure, and a 2.93 eV band-gap. This indicates that using materials from nature (green synthesis) is an effective route for the generation of semiconductor nanoparticles, with average sizes smaller than 100 nm. Furthermore, the use of ZnO NPs contributes directly to the worldwide problem of water pollution. The application of ZnO NPs present excellent photocatalytic properties, managing to degrade 100% of the MB organic dye in 60 min. In addition, they was able to degrade 85% of MO in 180 min, and 92% of RhB in 180 min. The range of applications of these nanoparticles could be very wide, considering that they are excellent catalysts in the elimination of contaminants present in water. ZnO NPs biosynthesized with *Capsicum annuum var. Anaheim* could be employed in removing various pollutants present in rivers, streams, and seas around the world.

## Figures and Tables

**Table 1:** Results of Rietveld refinement for the ZnO structure.

Phase	Crystal Structure	Space Group	Latice Parameter	ε (%)	Density (g/cm^3^)	Cell Volume (Å^3^)	R Factors (%)
Zincite	Hexagonal	P 63 m c	a = 3.25 Å b = 3.25 Å c = 5.21 Å c/a = 1.60 α = 90° β = 90° γ = 120°	0.379	5.65	47.81	R_e__xp_ = 6.97 R_p_ = 15.97 R_wp_ = 20.10 GOF = 2.88

**Table 2:** Photocatalytic activity of ZnO nanoparticles from some literature reports.

Year	NPs	Synthesis	NPs size (nm)	Pollutant	Degradation Time	Reference
2021	ZnO	*Green synthesis*	40	MB	100% in 60 min	This work
2021	ZnO	*Green synthesis*	40	MO	85% in 180 min	This work
2021	ZnO	*Green synthesis*	40	RhB	92% in 180 min	This work
2020	ZnO	*Green synthesis*	50	MB	55% in 200 min	[26]
2020	Ag/ZnO	*Amino acid assisted synthesis*	~20–50	MB	85 in 210 min	[75]
2020	ZnO	*Green synthesis*	30–50	MB	92% in 150 min	[76]
2020	Nanoflowers ZnO	Precipitation method	20–35	MO	100% in ~240 min	[77]
2020	ZnO:CuO	*Green synthesis*	~20–30	MO	45% in 60 min	[78]
2020	Au-ZnO	*Green synthesis*	33	RhB	74% in 180 min	[62]
2020	ZnO	*Green synthesis*	60–120	RhB	85% in 140 min	[79]

## Data Availability

Not applicable.
